# Experiencing literature on the e‐reader: the effects of reading narrative texts on screen

**DOI:** 10.1111/1467-9817.12337

**Published:** 2021-01-31

**Authors:** Annika Schwabe, Lukas Brandl, Hajo G. Boomgaarden, Günther Stocker

**Affiliations:** ^1^ Department of German Studies University of Vienna Vienna Austria; ^2^ Department of Communication Science University of Vienna Vienna Austria

**Keywords:** digital reading, e‐reader, reading comprehension, literary reading, fiction reading

## Abstract

**Background:**

The digitalisation of literature is proliferating, and the increasing spread of digital reading devices and the availability of digital texts is likely to make *books on screen* a lasting phenomenon, but little attention has been paid to the consequences of digitalisation for the experience of narrative fiction. While on the one hand, reading literature on a digital reading device might trigger a superficial processing of the text, and problems regarding orientation within the narrative, the awareness of reading a literary text might, on the other hand, lead to more in‐depth and complex processing, independent of reading medium. This study examines whether the reading performance and the emotional and cognitive experiences of the reception of a literary text vary between reading a printed book or an e‐reader.

**Methods:**

Using a between‐subjects experimental design, 207 participants read the beginning of a novel either in a printed book or on an e‐reader. They then completed a reading comprehension test and questionnaires about their cognitive and emotional experiences.

**Results:**

Overall, the results do not suggest the clear superiority of either of the two reading media. Neither reading speed nor reading comprehension differed significantly between the two groups. Even though a broad range of reading experiences was measured, neither cognitive nor emotional reading experiences differed significantly between the groups.

**Conclusion:**

An e‐reader does not affect either reading performance or cognitive and emotional experience of reading a narrative text, compared with a printed book.

## Highlights

*What is already known about this topic*

- Different reading media have different affordances.
- In general, reading comprehension of narrative texts does not differ between digital and paper reading.
- There is almost no empirical research into the effect of digital reading devices on reading dimensions other than comprehension.

*What this paper adds*

- Extensive research into the emotional and cognitive reading dimensions of different reading media when reading a complex literary text.
- There are no differences between an e‐reader and a printed book in subjective emotional and cognitive reading experiences during reading.
- The objective reading performance (reading comprehension and reading speed) depends very little on the reading medium.

*Implications for theory, policy or practice*

- E‐readers do not differ in most reading dimensions from the printed book when reading a narrative text.
- Neither the printed book nor the e‐reader is superior to the other one when reading a narrative text.
- E‐readers are better than their reputation.

Digital reading has become increasingly important across genres and text types. Reading the news on digital media has become commonplace over the past two decades (Thurman & Fletcher, 2019), and the consumption of e‐books may remain a permanent phenomenon. Statistics related to reading as leisure show that, for instance, more than 32 million e‐books, excluding textbooks, were sold in Germany in 2018, which translates to about 5% of the total book sales (Börsenverein des Deutschen Buchhandels, 2019). In the United States in 2016 and 2017, e‐books made up approximately 20% of all book sales (Statista, 2018). Such a fundamental change in the way people read books, that is, on which medium, coupled with the popular assumption that the medium has an effect on the reception of information, begs the question of whether reading on screen involves different reading experiences than reading printed books.

According to the central theses of cultural studies and phenomenological theory, different reading media go hand in hand with different reading behaviours and different reading experiences, due to the varying interfaces and affordances of the reading devices and different multisensory perceptions and haptics linked to the reading medium (Chartier, 1995; Mangen, 2016). Readers are never confronted with an abstract, immaterial text, but rather with concrete objects that co‐determine their understanding of the written text.

A host of prior studies have empirically addressed this matter, largely focusing on the potential differences in the actual comprehension of texts between screen and paper reading. Summing up this literature, in their recent meta‐analyses, Kong, Seo, and Zhai (2018), Delgado et al. (2018) and Clinton (2019) looked at the relationship between reading medium and reading comprehension and found a significant paper‐based reading advantage in comparison to digital reading. People who read on paper were better overall at recalling information that was provided in the text. The vast majority of studies addressing differential outcomes of reading in print versus reading on screen has focused on informational texts such as school books or expository texts and on the effects on a reader's understanding of such texts. Apart from a number of notable exceptions (e.g., Guarisco, Brooks, & Freeman, 2017; Haddock, Foad, Saul, Brown, & Thompson, 2019; Mangen, Olivier, & Velay, 2019), the literature so far has neglected (1) the reception of narrative and fictional e‐books for leisure reading and (2) the potential effects of digital reading on a broader conception of the reading experience. While informational texts may be primarily concerned with generating understanding of the information presented, we argue that reading literature during leisure time is about more than just text comprehension.

Despite the increasingly widespread use of screens to consume literary texts, little is known about the possible effects of leisure reading on digital devices on reading performance, and especially on aspects of the reading experience. This study addresses these gaps by experimentally investigating the consequences of screen versus print reading of high‐brow literature on a multitude of dimensions of the reading experience. The study focuses on e‐readers as the digital substrate because they are the most common medium for reading e‐books (PricewaterhouseCoopers, 2017).

The current study builds on the insights from the previous literature, but adds to these in important ways to investigate the impact of reading narrative texts on different media. First, we not only focused on performance‐based facets, such as reading comprehension and reading speed, but importantly considered a great range of other aspects of the multidimensionality of literary reading. These include, among other things, absorption in the text, becoming emotionally involved, evaluating text characteristics, and effects on the current mood. Second, to enable a common literary reading experience, we used the beginning of a novel with a relatively complex structure and high ambiguity as the stimulus. Third, we provided a natural haptic experience with the original printed book, instead of printed‐out papers, and the original e‐book on an Amazon Kindle. The present study thus provides a highly relevant and comprehensive empirical perspective on the effects of reading literature on screen.

### Theoretical framework

The literature currently provides different theoretical perspectives and assumptions about why the reading experience might be expected to differ between print and screen reading. Mangen and van der Weel (2016) constructed a transdisciplinary framework describing reading as a complex process involving ergonomic, attentional, cognitive, emotional, and phenomenological dimensions, and as depending on historical, cultural, medial, textual, individual, and situational factors. Reading involves interaction with a device with specific interface affordances, and reading entails physical (in particular, manual/haptic) interaction with the reading medium (Mangen & van der Weel, 2016). Recently, Spence (2020) referred to the multisensory experience of handling and reading books, highlighting the emotional and nostalgic associations that are triggered while interacting with a physical book and the lack of nonvisual senses in the reader's experience when reading digitally.

One assumption is that crucial sensorimotor cues, such as the tangibility, the text length overview, or where a piece of specific information is located within a book and where it is placed on a page, are limited when the text is displayed on a screen. A printed book is a three‐dimensional reading medium, whereas an e‐book is only displayed in two dimensions. This might lead to difficulties understanding the structure of the text and creating a cognitive map of the text. Crucial contextual information and navigational mechanisms might be missing using an e‐book (Li, Chen, & Yang, 2013). Mangen et al. (2019) also offer empirical evidence for this claim. While they did not find a difference in overall reading comprehension between reading a narrative text digitally and reading the same text on paper, digital reading was associated with a poorer comprehension of chronology and reduced ability to locate specific events in the text.

A crucial aspect of research into the digitalisation of reading involves outcomes that can be classified as reading performance, considering the comprehension of text and the speed in which text is read. Another assumption about paper‐based reading advantages from this perspective is that digital reading media impede in‐depth processing by triggering lower level processing habits such as quick scanning or skimming a text (Lauterman & Ackerman, 2014; Kaufman & Flanagan, 2016). Digital media, as such, may thus prompt people to process texts differently simply by virtue of them being digital. Further and relatedly, the so‐called cognitive control system is of importance to the reading process. This cluster of knowledge structures and processes triggered by outside information or by other cognitive goals regulates attitudes towards a specific situation (Zwaan, 1993). Applied to reading, this suggests that the material that carries the text would activate certain types of knowledge and processing routines that may affect the actual processing of the text. This indicates that approaching a literary text on a digital medium might result in faster reading time and, therefore, in poorer reading comprehension than reading in print.

Clinton's (2019) meta‐analysis did not confirm that reading speed (as an indicator of skimming or in‐depth processing) differs between digital and printed reading media, and other studies not included in their analysis have come to similar conclusions. For skilled readers, there does not seem to be a difference in reading speed on a computer screen (Köpper, Mayr, & Buchner, 2016; Çınar, Doğan, & Seferoğlu, 2019) or an e‐reader (Siegenthaler, Wurtz, Bergamin, & Groner, 2011; Cavalli et al., 2019) compared with reading the same text in print. Of course, this largely contradicts the assumptions mentioned before; however, more specific research on reading performance, especially using complex narrative texts, is necessary to determine the effects of the medium concerning leisure reading. Therefore, and taking into account the inconclusiveness of prior empirical findings, we ask

*Does reading* performance *(reading speed and different aspects of reading comprehension) depend on the reading medium when reading a narrative text?*

Reading performance in terms of speed and text comprehension is, however, by no means a comprehensive account of the reading experience of literary texts. Performance‐based indicators lack the peculiarities of reading fiction, such as the emotional and experiential aspects of the reading experiences. Reading narrative fiction is a complex process, which differs from the reading experience of nonliterary texts. What remains important for the understanding of the literary reading process is – predominantly pointed out by theorists of reader‐response criticism (Tompkins, 1980) – the notion of the reader's dynamic activity throughout the text, and the realisation that the meaning of a literary text is not a stable and fixed entity, but that the variable result of an individual construction process (Iser, 1978; Gerrig, 2011). This construction process consists of the interplay between different levels, and it is determined by the text, the concrete reader, the reading environment and, which we claim is also fundamental, by the reading device. One key element of the literary experience is that readers shift into the fictitious world that the text designs, which Gerrig (1993) popularised as the term *transportation*, the impression of leaving the real world and visiting narrative worlds. *Immersion* and *absorption* are alternative concepts related to transportation and used synonymously in the relevant academic discourse. Another crucial dimension of the specific experience with literary texts is emotional involvement in the narrative, which is strongly associated with narrative coherence and absorption, combining affective and cognitive dimensions (Busselle & Bilandzic, 2009).

Mangen and Kuiken (2014) noted that, to their knowledge, they were the first to empirically study the effect of the reading medium on transportation and empathy. Still today, the body of empirical literature is sparse and does not come to a uniform conclusion about the differences in reading experiences when reading digitally or on paper. On the one hand, the phenomenological characteristics and the handling of the reading medium must not be distracting, so that the reader can easily be transported into the story (Mangen & Kuiken, 2014). On the other hand, when the reader is transported into the story, the actual reading medium could become irrelevant, as does the rest of the physical surroundings (Kuzmičová, 2014).

Mangen et al. (2019) and Lange (2019) did not find an effect of the reading medium on transportation, but the results found by Haddock et al. (2019) were inconclusive. They presumed that the read narrative moderates the effect of the reading medium. They found that the reading medium does not affect transportation when reading a modern story, but that transportation is disturbed when reading a traditional story digitally. Further, while the general interest in the text does not seem to depend on the reading medium (Grimshaw, Dungworth, McKnight, & Morris, 2007; Moyer, 2011), the effect of the reading medium on empathy and theory of mind are also ambiguous (Mangen & Kuiken, 2014; Guarisco et al., 2017). Kaakinen et al. (2018), however, concluded that far too little is known about the emotional and cognitive aspects of digital reading.

Considering the objectives of the integrative framework by Mangen and van der Weel (2016) and the requirement of theoretical perspectives from different disciplines and with the aim of understanding the digital literary reading process in its entire multidimensional complexity, we therefore consider those dimensions of the literary experience, which are the most commonly mentioned and examined in psychology, literary studies and educational science. In addition to transportation (we use the term absorption in our analyses) and empathy, the identification and mental visualisation of a fictitious world, fictitious characters and their actions are also basic conditions for the literary experience (Iser, 1978; Spinner, 2016). Apart from emotions that arise during the reading process, reading fiction can also have an effect on emotions after reading (Mar, Oatley, Djikic, & Mullin, 2011).

Readers can enjoy specific aesthetics in a literary text, such as its rich descriptions or dense metaphors. They might also compare it with other books they have read, make inferences about its historical or political context, their own personal situation or other dimensions beyond the concrete story and its characters. Vorderer (1994) refers to such reception of narratives as the *analysing reception*, in which different aspects of the text are analysed and evaluated from a distant stance.

Given the lack of prior research into some of these important dimensions and the inconclusiveness of extant empirical research, we ask

*Does the reading medium affect various dimensions of the reading experience?*

## Methods

We addressed the two research questions formulated earlier by means of an experiment in which participants were reading the same text in a printed book or on an e‐reader.

### Sample

Given that our research interests are primarily relevant to a population that habitually reads literature, the participants were recruited via posters and flyers at bookstores, libraries, public literature reading venues, and via select social media channels. The participants received €20 remuneration for their participation in the experiment. The original sample consisted of 211 participants, but we had to exclude three participants because they did not stop reading at the endpoint and read further than instructed and one participant due to insufficient language skills. The final sample therefore consisted of 207 German‐speaking participants (whole sample: 19–72 years, mean age = 29.96, 168 female, 38 male, one nonbinary; print condition: 19–68 years, mean age = 29.57, 85 female, 19 male; screen condition: 19–72 years, mean age = 30.35, 83 female, 19 male, one nonbinary).

### Setting and procedure

To provide a reading setting that was as natural as possible, we set up a reading chair with a reading light in which the participants sat while reading the text and were allowed to eat biscuits and drink water during reading. As part of the between‐subject design, the participants were randomly assigned to read either the original printed hardcover book or the original e‐book on a Kindle Paperwhite (fourth gen.). The Kindle Paperwhite has a 6‐inch touch display with 300 ppi and five light‐emitting diode background lights. To obtain as much external validity as possible, participants in the Kindle condition were allowed to change the font size and luminance according to their needs. Because fonts can affect the evaluation of a text's content (Kaspar, Wehlitz, von Knobelsdorff, Wulf, & von Saldern, 2015) and to better control the setting, we did not allow the participants to change the actual font type. The font was set to Bookerly, which is the Kindle's factory setting. In the printed version of the text, the font size was 10 pt and the font type was Janson. Before and after reading, the participants answered an online questionnaire on a desktop computer.

### Reading material

Unlike many studies, which have mostly used short stories as stimulus material, we examined the reading experience with a complex narrative and within a longer reading time frame. We chose the first 20 pages of the high‐brow novel ‘Schöne Freunde’ (*Nice Friends*) by the contemporary Austrian author Arno Geiger (2002). We chose this text because it is a relatively unknown novel by a highly acknowledged and much‐read author in Austria and Germany. It is a complex fictional text characterized by an unreliable narrator, a nonchronological order of the narrated events and a very characteristic poetic style: a curious boy, the gatekeeper of a mine, tells a quirky story about an ordinary, bourgeois village, the love affairs of its inhabitants and his search for a vanished love. The starting point of the novel is an indeterminate mine accident, which forces the people to leave the village.

### Reading comprehension

A team of two literary scholars and one psychologist developed an instrument to measure different aspects of reading comprehension focused on the specific literary features of the chosen text. The participants had to name the author, the title of the book and the title of the chapter they read to explore their knowledge about the paratextual information of the text. Further, according to the situation model by Zwaan, Langston, and Graesser (1995), the participants had to list every person, animal, and place mentioned in the text that they could remember. In order to measure their comprehension of the chronology, participants had to put a list of ten events into the order in which they were mentioned in the text (‘discourse’ in narratology) and in which they happened in the story (‘histoire’ in narratology). Finally, participants had to answer 20 detailed multiple‐choice questions about the first part of the text, the middle part, the end part and the whole text. The multiple‐choice questions had seven answers, with one of them being ‘I don't know’ and a varying number of true and false possibilities.

### Reading speed

We measured the reading time with a standard stopwatch.

### Cognitive and emotional reading experience

To explore the reading experience extensively, we used two different scales to measure 20 different dimensions of the cognitive and emotional reading experience after reading the text. We used the German ‘Aspekte des Leseerlebens’ scale (aspects of the reading experience) by Appel, Koch, Schreier, and Groeben (2002), which consists of 77 items covering 14 factors: *focus of attention*, *being absorbed in the text*, *imaginability*, *spatial presence*, *end of reception*, *excitement*, *emotional involvement*, *general reading pleasure*, *identification*, *parasocial interaction*, *cognitive involvement*, *thematic interest*, *analysing reception* and *ease of cognitive access*. The participants rated on a 6‐point Likert scale how far they agreed with the statements. The Cronbach's α of these dimensions varied in our sample between .68 and .92.

We also used the ‘Narrative and Aesthetic Feelings’ scale by Koopman (2015). We translated the 26 original Dutch 7‐point Likert items into German. The factors of this scale were *sympathy/empathy*, *identification*, *absorption*, *empathic distress*, *attractiveness* and *foregrounding*. The Cronbach's α of these dimensions varied in our sample between .7 and .92.

### Mood changes

To measure changes in mood, we used the German translation of the ‘Positive and Negative Affect Schedule’ by Krohne, Egloff, Kohlmann, and Tausch (1996). To ensure possible mood changes occurred as a result of the experience of reading this particular text digital or on paper and not because of other factors, participants filled out the questionnaire directly before and a second time directly after reading the text. The Cronbach's α varied between .8 and .87.

### Control questions

To determine whether the participants had heard of the author Arno Geiger without revealing that they would read a text by this author, we asked them to fill out the German version of the ‘Authors Recognition Test’ version A (Grolig, Tiffin‐Richards, & Schroeder, 2020) before reading the text, which we extended to include 10 Austrian authors, one of whom was Arno Geiger, and five random Austrian names. Almost half of the participants (44.9%) had heard of the author Arno Geiger before reading the text. We also asked the participants after their reading if they had read the text before. All participants were reading the text for the first time during the experiment.

### Data analysis

We analysed our data as planned and described in our preregistration. 1 We had two kinds of open recall questions. We analysed the items for which the participants had to list as many persons, animals and locations as they could remember by summing up all answers to one summary score. We then used this summary score in a two‐tailed two‐sample *t* test with the reading medium as the group variable. We calculated a summary score of the three dichotomous items (right or not right) where participants had to name the author, title of the book and title of the chapter. Because we did not know whether the items had the same level of difficulty and because with only three items, there were only four different outcomes, we treated the data as ordinal and used a two‐tailed Mann–Whitney *U* test with the medium as group variable. Exploratory and additional to our preregistration, we also conducted three Chi^2^ tests for each of the paratextual items separately.

We used pairwise absolute row differences (Bartok & Burzler, 2020) for the two chronology sorting items, using the correct answers as references and two‐tailed two‐sample *t* tests with the pairwise absolute row differences scores as the dependent variable and reading medium as the independent variable. We calculated a sum score for each type of multiple‐choice question (about the beginning, middle, end and the whole text) and used them in a single‐factor multivariate ANOVA with reading medium as the independent variable. To check for possible main effects, we conducted two‐tailed two‐sample *t* tests with reading medium as the group variable for each type. We carried out two mixed ANOVAs to analyse the interaction between reading medium and positive and negative affect separately for the changes in positive and negative affect and two two‐tailed paired sample *t* tests for each experimental condition separately for the main effects. Further, we performed for each of the 20 reading experience factors and for the reading speed a two‐tailed two‐sample *t* test with reading medium as the group variable.

We set the level of significance to *p* < .05

## Results

### Reading performance

To answer RQ1, we first considered the effects on the various types of measures that relate to the reading performance, including speed but, more importantly, various and detailed aspects of comprehension. The overall knowledge about the paratextual information was significantly better when participants read the text in the original printed book (*U* = 4106.5, *p* = .002). Further, the participants reading print were significantly better at naming the title of the book (*X^2^(1, N = 207) = 29.8, p* < .001), but not significantly better at naming the author (*X^2^(1, N = 207) = 3.04, p* = .08), and they were significantly worse at naming the chapter title than the participants reading the e‐book (X^2^(1, N = 207) = 5.5*, p* = .02). Participants performed the same in both conditions regarding what we refer to as situation model. Neither group was better at listing mentioned persons, animals and places (*t*(205) = 1.22, *p* = .23). In the two chronology tasks, where the participants had to sort the events into the order in which they were mentioned and in which they happened in the story, the results of the participants did not significantly differ between the two experimental groups (order mentioned in the text: *t*(202) = −1.12, *p* = .27; order happened in the story: *t*(199) = −1.77, *p* = .18). There was also no significant difference in the number of correct multiple‐choice questions in general, *F*(4, 202) = 1.69, *p* = .16. There was no effect on the number of correct answers in the multiple‐choice questions about the whole text, the middle part, and the end (whole text: *t*(205) = 1.03, *p* = .3; middle part: *t*(205) = 0.63, *p* = .53; end: *t*(205) = 0.8, *p* = .43); however, participants reading in a printed book scored higher in questions concerning the first part of the text (*t*(205) = 2.42, *p* = .02). Nevertheless, there was no significant difference in reading speed (*t*(205) = 0.45, *p* = .65). In sum, our results regarding RQ1 suggest only few differences in reading performance based on the range of indicators included in our study. We found differences between screen and print readers only for some paratextual information and regarding a particular set of knowledge questions.

### Reading experiences

The differences between reading media in various aspects of the reading experience are described here to address RQ2. There was no significant difference in mood change, *F*(1, 205) = 0.3, *p* = .58, for either positive or negative affect between the two reading media (positive affect, *F*(1, 205) = 0.42, *p* = .52; negative affect, *F*(1,205) = 1.93, *p* = .166). Reading the text did not significantly change the negative affect in either of the groups, but positive affect decreased in both groups (print: *t*(103) = 4.11, *p* < .001; digital: *t*(102) = 4.6, *p* < .001).

On its own, the reading medium also did not have an effect on any emotional and cognitive reading dimension (Table 1). We did not find significant group differences for any of the 20 dimensions that were included, and overall, the results did not even approach conventional levels of significance. 2

**Table 1:** Effect of the reading medium on emotional dimensions of reading

	*df*	*t*	*MD* (*SE*)	*MD* Lower CI (95%)	*MD* Upper CI (95%)	*M* (*SD*) print	*M* (*SD*) digital	*p*
Aspects of the reading experience								
Focus of attention	205	−1.4	−1.7 (0.84)	−2.82	0.484	15.38 (5.85)	16.54 (6.21)	.16
Being absorbed in the text	205	1.18	0.88 (0.75)	−0.59	2.35	15.46 (5.17)	14.58 (5.56)	.24
Imaginability	205	0.2	0.16 (0.85)	−1.4	1.72	18.38 (5.6)	18.22 (5.81)	.84
Spatial presence	205	0.31	0.26 (0.85)	−1.41	1.94	15.95 (5.76)	14.69 (6.45)	.76
End of reception	205	0.39	0.21 (0.54)	−0.86	1.28	12.47 (4.00)	12.26 (3.79)	.7
Excitement	205	1.06	0.69 (0.65)	−0.6	1.97	16.17 (4.80)	15.49 (4.57)	.29
Emotional involvement	205	1.71	1.26 (0.74)	−0.2	2.72	16.04 (5.56)	14.78 (5.04)	.09
General reading pleasure	205	0.24	0.23 (0.93)	−1.6	2.05	18.77 (6.95)	18.54 (6.34)	.81
Identification	205	−0.86	−0.97 (1.12)	−3.17	1.24	28.41 (7.69)	29.38 (8.37)	.39
Parasocial interaction	205	−0.38	−0.24 (0.65)	−1.52	1.03	11.14 (4.54)	11.39 (4.75)	.71
Cognitive involvement	205	0.2	0.14 (0.71)	−1.26	1.54	15.36 (4.72)	15.21 (5.47)	.84
Thematic interest	205	−1.12	−0.77 (0.69)	−2.13	0.59	13.40 (4.96)	14.17 (4.97)	.27
Analysing reception	205	0.48	0.46 (0.97)	−1.45	2.38	30.2 (7.12)	29.74 (6.82)	.63
Ease of cognitive access	205	−0.38	−0.3 (0.77)	−1.82	1.23	15.39 (5.47)	15.69 (5.63)	.7
Narrative and aesthetic feelings								
Sympathy/empathy	197.65	0.01	0.01 (1.04)	−2.03	2.05	26.10 (6.73)	26.09 (8.11)	.99
Identification	197.72	0.3	0.16 (0.53)	−0.89	1.21	8.31 (4.20)	8.15 (3.43)	.76
Absorption	205	0.62	0.62 (1)	−1.34	2.58	18.99 (7.06)	18.37 (7.27)	.53
Empathic distress	205	0.02	0.02 (0.81)	−1.58	1.61	9.12 (5.69)	9.10 (5.95)	.98
Attractiveness	205	−0.12	−0.11 (0.89)	−1.85	1.64	18.00 (6.50)	18.11 (6.23)	.9
Foregrounding	205	1.1	0.74 (0.67)	−0.58	2.06	15.49 (4.84)	14.75 (4.79)	.27

CI, confidence interval

## Discussion

Although the importance of tablets, smartphones and e‐readers as reading media is increasing, most research has continued to focus on computer screens as the reading device. Research is also limited in the types of text it uses and has mostly focused on informational texts (Delgado et al., 2018). Both circumstances limit the generalisability of the insights of these studies to the reality of digital literature reading. The present study addressed this shortcoming and explicitly focused on the difference between reading a narrative high‐brow text on an e‐reader and in a printed book. Despite a thorough experimental design, the findings do not suggest a significant difference overall between reading the text in a printed book and reading it on an e‐reader.

The few exceptions arose only with regard to reading *performance* indicators. Participants in the print condition seemed able to remember the paratextual information better than the participants in the digital condition. Nonetheless, when looking at the items separately, the picture is not as clear. The participants reading in the original printed book were better at remembering the title of the book probably because they were able to look at the book cover again after reading it when closing the book and giving it back to the test administration. At the same time, there was no difference between the groups in remembering the name of the author. We cannot explain why the participants reading the e‐book outperformed participants reading the printed version in naming the title of the chapter. In both conditions, the chapter title was on the same page as the first paragraph of the text and equally distinct from the rest of the text.

In line with Cavalli et al. (2019) but contrary to Mangen et al. (2019), both experimental groups performed equally well in the chronology of the story test. There was also no difference in participant knowledge of when events were described in the text. Mangen et al. (2019) used a text where the story was told in chronological order. In contrast to Mangen et al. (2019), we used a text with many flashbacks, which might be more difficult to process than a completely linear story. The construction of a cognitive map of the story might be more difficult in general, regardless of the reading medium.

Neither of the two experimental groups exceeded the other in listing characters, places, or animals, contrasting with the results of Margolin, Snyder, and Thamboo (2018). In general, the participants in the two conditions did not differ in answering detailed multiple‐choice questions. The only exception was the questions about the first part of the text. Participants who read the printed version were able to answer these questions significantly better than the participants who read the digital version, supporting findings by Mangen et al. (2019). They suggest that the haptic clues of the physical book might support the formation of a better cognitive map, which helps with remembering information that was acquired less recently as the information about the middle and the last part of the text. Again in line with Cavalli et al. (2019), we did not find a significant difference in reading speed.

Despite these few differences in reading performance, there was no discernible effect of the medium on the literary *experience*, which is possibly the more important part of leisure literature reading. Even though our analysis was very comprehensive and extensive in terms of the number of different dimensions of the literary experience, we did not find that the reading medium had an effect on any of these. None of the factors described by Appel et al. (2002) and Koopman (2015) were significantly affected by the reading medium. Changes in mood were also not dependent on the medium. These results mostly coincide with the sparse available literature on the impact of an e‐reader on emotional and cognitive reading dimensions (e.g.,Moyer, 2011; Mangen et al., 2019), yet provide a more comprehensive and elaborate account in comparison.

The results are perhaps not surprising. The affordances of an e‐reader are more similar to the affordances of a printed book than to the affordances of a computer, tablet, or smartphone. While multimedia devices can also be used for a broad range of different activities, printed books and e‐readers are designed simply for the purpose of reading. They might not trigger the digital media effect of less in depth‐processing as described by Lauterman and Ackerman (2014). The eye movements while reading on e‐ink displays are also similar to those while reading in print. Compared with liquid crystal displays, the font‐background‐contrast of an e‐reader can be similar to paper, and e‐ink provides good visibility in varying light conditions. The legibility of an e‐reader may therefore not be inferior to print. If anything, the legibility of e‐readers might be better than those of print because the font size can be individually optimised (Siegenthaler et al., 2011).

Both Delgado et al. (2018) and Clinton (2019) also used text type as a moderating variable and found that the superiority of reading in print is only present when informational texts were read and not when narrative texts were read. With narrative texts as stimuli, the meta‐analyses both found no difference between reading on screens and on paper. Knowledge about the effects of digital reading of informational texts therefore cannot be directly transferred to the reading of narrative texts. Simply the assumption of reading a literary text triggers a specific control system in the minds of readers, which regulates the comprehension of the text (Zwaan, 1993). Although the same text is read, there are differences in neuronal processing with different paratextual framing, depending on whether readers believe they are reading a factual or fictional text (Altmann, Bohrn, Lubrich, Menninghaus, & Jacobs, 2014). When reading a literary text, the eventual importance of every described aspect is primarily unknown, and the meanings of the total picture unfold during the reading process. Literary reading therefore involves more attention to the textbase, the surface structure and a relatively good memory of verbatim information (Zwaan, 1993; Rosebrock, 2018). The immersion might lead to less awareness of the real world (Green & Brock, 2000), and therefore, the reading medium could fade into the background. It is possible that the more text‐focused reading strategy while reading narratives means that the reading medium loses importance.

Our study is not without limitations. Although our results strongly suggest no difference between reading a narrative text on an e‐reader and in print, the insights may not travel to digital reading in general. Further, we chose to let participants set the font size to their preference and did not align the digital and the printed versions of the text, so that the differences between reading on an e‐reader and in a printed book, might have actually arisen due to the difference in words per page and not due to different reading media. This has not yet been empirically explored with a novel as stimulus, but Hou, Rashid, and Lee (2017) were able to show that reading comprehension and immersion in reading a comic were the same between the digital and the printed versions when they had the same amount of panels per page. Reading comprehension was worse when there was only one panel per page compared with the other conditions. Further, in our experiment, the situation, as well as the chosen text, were artificial because both were completely chosen by us, while leisure reading is, in most cases, an intrinsically motivated action. The controlled lab setting might also trigger a mindset similar to an exam situation because the participants know they will be tested after reading. Most external or medium‐specific distractions are eliminated or controlled for in a lab setting, which also limits the generalisability of the results. Other methods, such as mobile experience sampling in a natural setting, might thus be worth exploring when studying leisure reading.

Future research should continue to use the authentic printed book as a comparison medium for different digital reading devices. In spite of the importance of mobile reading in general, the difference in reading a narrative text in print and reading on a smartphone has to our knowledge barely been explored. Future research should resume investigation of the importance of individual differences for the digital reading process. Different levels of working memory, as well as personality, were shown to affect digital reading (Hou, Wu, & Harrell, 2017; Margolin et al., 2018), but the studies have not been replicated yet. Exploring digital reading in large and diverse samples could therefore shed light on the differences in digital fiction reading.

Probably the biggest research gap within this field is the actual difference between reading a narrative and an informational text on screen. Studies using an informational text show the superiority of reading in print over reading on screen, but studies using a narrative text fail to show this effect (Clinton, 2019; Delgado et al., 2018). Despite our theoretical assumptions, the reason for this is still unclear and needs further investigation, with an accurate focus on the specific textual features and particular effects of literary texts.

## Conclusions

While other studies suggest that the reading medium might have an effect on the reading performance, we did not find such a coherent effect for printed books vs e‐readers. Most of our results did not reach significance, indicating that the reading performance was not significantly dependent on the reading medium and did not differ between reading the same high‐brow literary text in the original printed book and the original e‐book on a Kindle. Further, we did not find a single significant result suggesting that the emotional and cognitive literary experiences during reading might be positive or negative due to an e‐reader in comparison with the printed version. Our results cannot be simply transferred to digital reading in general, however, due to the different affordances of e‐readers and other digital reading devices. Because literary reading takes place more and more not only in the printed book or on the e‐reader, the important question is still which medium triggers which cognitive control system due to its specific affordances and variety of applications. More research concerning literary reading with tablets and especially smartphones would therefore be worthwhile in order to understand the overall consequences of digital reading.

## Conflict of interest

We have no known conflict of interest to disclose.

## Funding

The results presented here are part of the ‘Books on Screen’ project P 31723‐G30, funded by the Austrian Science Fund (FWF).

## Data availability statement

The data that support the findings of this study are available from the corresponding author upon reasonable request.

## Footnotes

1
Preregistration: Schwabe, A., Brandl, L., Stocker, G., & Boomgaarden, H. (2019, December 1). Books on screen (https://doi.org/10.17605/OSF.IO/VQKC4).

2
In addition, as described in our preregistration, we also performed analyses controlling for reading speed as an effect on the association of reading medium and reading experiences; however, the reading speed did not affect the relationship between reading medium and reading dimensions. Additionally to our preregistration and as a response to the mostly nonsignificant results, we also conducted tests of equivalence using the r package TOSTER (version 0.3.4; Lakens, 2017). We set the lower and upper bounds of equivalence to *d* = |0.5| because that is the size of an effect where one would become aware of an average difference (Cohen, 1988). The results showed that for all comparisons that were not significant in the primary analyses, the two reading conditions are equivalent to the level of *d* = 0.5. Further, to estimate the precision of the effect sizes found, we bootstrapped the sample and repeated the analyses (*n*_Samples_ = 1,000). The bootstrapping analyses replicated the results as discussed earlier and thus provides further evidence for the robustness of the findings and showed that the results provided earlier are robust against sampling distributions. Tables of the tests of equivalence and bootstrapping analysis are shown in Appendices A and B. Further specific results of the analyses are available on request from the authors.

## Equivalence TOST test

**Table:**

		*df*	*t*	*p*
Aspects of the reading experience				
Focus of attention	*t* Test	205	1.39	.16
	TOST upper bound	205	−2.2	.01
	TOST lower bound	205	4.99	<.001
Being absorbed in the text	*t* Test	205	−1.18	.24
	TOST upper bound	205	−4.78	<.001
	TOST lower bound	205	2.42	<.01
Imaginability	*t* Test	205	−0.2	.84
	TOST upper bound	205	−3.8	<.001
	TOST lower bound	205	3.4	<.001
Spatial presence	*t* Test	205	−0.31	.76
	TOST upper bound	205	−3.91	<.001
	TOST lower bound	205	3.29	<.001
End of reception	*t* Test	205	−0.39	.7
	TOST upper bound	205	−3.98	<.001
	TOST lower bound	205	3.21	<.001
Excitement	*t* Test	205	−1.06	.29
	TOST upper bound	205	−4.65	<.001
	TOST lower bound	205	2.54	<.01
Emotional involvement	*t* Test	205	−1.71	.09
	TOST upper bound	205	−5.31	<.001
	TOST lower bound	205	1.89	.03
General reading pleasure	*t* Test	205	−0.24	.81
	TOST upper bound	205	−3.84	<.001
	TOST lower bound	205	3.35	<.001
Identification	*t* Test	205	0.86	.39
	TOST upper bound	205	−2.73	<.01
	TOST lower bound	205	4.46	<.001
Parasocial interaction	*t* Test	205	0.38	.71
	TOST upper bound	205	−3.22	<.001
	TOST lower bound	205	3.97	<.001
Cognitive involvement	*t* Test	205	−0.2	.84
	TOST upper bound	205	−3.8	<.001
	TOST lower bound	205	3.4	<.001
Thematic interest	*t* Test	205	1.12	.27
	TOST upper bound	205	−2.48	<.01
	TOST lower bound	205	4.71	<.001
Analysing reception	*t* Test	205	−0.48	.63
	TOST upper bound	205	−4.08	<.001
	TOST lower bound	205	3.118	<.01
Ease of cognitive access	*t* Test	205	0.38	.7
	TOST upper bound	205	−3.21	<.001
	TOST lower bound	205	3.98	<.001
Narrative and aesthetic feelings				
Sympathy/empathy	*t* Test	205	−0.01	.99
	TOST upper bound	205	−3.61	<.001
	TOST lower bound	205	3.59	<.001
Identification	*t* Test	205	−0.3	.77
	TOST upper bound	205	−3.9	<.001
	TOST lower bound	205	3.29	<.001
Absorption	*t* Test	205	−0.62	.53
	TOST upper bound	205	−4.22	<.001
	TOST lower bound	205	2.97	<.001
Empathic distress	*t* Test	205	−0.02	.98
	TOST upper bound	205	−3.62	<.001
	TOST lower bound	205	6.57	<.001
Attractiveness	*t* Test	205	0.12	.9
	TOST upper bound	205	−3.48	<.001
	TOST lower bound	205	3.72	<.001
Foregrounding	*t* Test	205	−1.11	.27
	TOST upper bound	205	−4.71	<.001
	TOST lower bound	205	2.49	<.01
Reading comprehension (multiple‐choice questions)	*t* Test	205	−1.71	.08
	TOST upper bound	205	−5.3	<.001
	TOST lower bound	205	1.89	.03

*Note*. The lower bound is set to *d* = −0.5 and the upper bound to *d* = 0.5.

## Bootstrap for independent samples test

**Table:**

	*MD* (*SE*)	Bias	*MD* lower CI (95%)	*MD* upper CI (95%)	*p*
Aspects of the reading experience					
Focus of attention	−1.7 (0.82)	0.01	−2.82	0.36	.15
Being absorbed in the text	0.88 (.73)	0.004	−0.62	2.22	.24
Imaginability	0.16 (0.77)	−0.02	−1.36	1.71	.82
Spatial presence	0.26 (0.83)	−0.01	−1.41	1.94	.77
End of reception	0.21 (0.54)	0.001	−0.85	1.28	.71
Excitement	0.69 (0.65)	0.01	−0.57	2.0	.31
Emotional involvement	1.26 (0.75)	0.02	−0.17	2.69	.09
General reading pleasure	0.23 (0.94)	−0.01	−1.67	1.99	.8
Identification	−0.97 (1.12)	−0.14	−3.35	1.08	.41
Parasocial interaction	−0.24 (0.66)	0.36	−1.55	1.04	.72
Cognitive involvement	0.14 (0.73)	−0.01	−1.35	1.56	.85
Thematic interest	−0.77 (0.67)	−0.01	−2.12	0.56	.24
Analysing reception	0.46 (0.98)	−0.01	−1.46	2.44	.61
Ease of cognitive access	−0.3 (0.75)	−0.1	−1.78	1.28	.69
Narrative and aesthetic feelings					
Sympathy/empathy	0.01 (1.02)	−0.3	−2.02	1.87	.99
Identification	0.16 (.519)	−0.002	−0.87	1.17	.79
Absorption	0.62 (.97)	−0.01	−1.23	2.58	.53
Empathic distress	0.02 (.79)	0.02	−1.5	1.57	.98
Attractiveness	−0.11 (.87)	0.001	−1.85	1.61	.9
Foregrounding	0.74 (0.67)	−0.01	−0.54	2.13	.28
Reading Comprehension (Multiple‐choice questions)	.38 (.22)	−0.004	−0.07	0.81	.08

*Note*. Bootstrap results are based on 1,000 bootstrap samples.
